# Supplementary material for: WordCommentsAnalyzer: A windows software tool for qualitative research
Source: F1000Res. 2018 Sep 4;7:536. Originally published 2018 May 3. [Version 2] doi: 10.12688/f1000research.14819.2 (PMC6137411; doi:10.12688/f1000research.14819.2)
Supplement: Supplementary file 3 [file f1000research-7-17614-s0002.tgz › b2a79496-4ada-47c4-9682-a99ce867836d.zip!/Eight Iranian Health Journals/Salmand_Iranian Journal of Ageing _40.docx]

© Salmand_Iranian Journal of Ageing . This work is licensed under CC BY-NC 4.0 (https://creativecommons.org/licenses/by-nc/4.0/).

Volume & Issue: دوره 3 - شماره 2

Title: بررسی عوامل مؤثر بر سلامت خود ادراک سالمندان ایران

http://salmandj.uswr.ac.ir/article-1-88-fa.html

اهداف: این مقاله شناخت محدودیت فعالیت، محدودیت عملکرد و اختلال روانی، ویژگی‌های جمعیتی-اجتماعی (پایگاه اقتصادی‌اجتماعی، تحصیلات، وضع فعالیت، سن و جنس) در سالمندان و تاثیر آنان بر سلامت خود ادراک سالمندان است. همچنین برازش داده‌های سلامت سالمندان با مدل ارائه شده توسط جانسون و ولینسکی و ارائه مدل نظری و تحلیلی مناسب برای بررسی همزمان نقش متغیرهای ذکر شده از اهداف این پژوهش می‌باشد. مواد و روش‌ها: مطالعه توصیفی تحلیلی از نوع مقطعی داده های طرح ملی پیمایش وضعیت سلامت سالمندان در جمهوری اسلامی ایران در سال 1381 است و برای تبیین سلامت خود ادراک سالمندان از دو رهیافت سلامتی جمعیت و رویکردهای شاخصهای سلامتی استفاده شده است. یافته‌ها: تحلیل آماری نشان داد سالمندانی که محدودیت عملکرد، محدودیت فعالیت و اختلال روانی بیشتری داشته‌اند نسبت به کسانی که هیچ مشکل سلامتی نداشته‌اند سلامتی خود را ضعیف تر گزارش داده‌اند و مشاهده شد افراد طبقات اجتماعی پایین‌تر که دارای درآمد کمتر و سطح تحصیلات پایین‌تر هستند نسبت به افراد از طبقه اجتماعی بالاتر که درآمد بیشتر و سطح تحصیلات بالاتری دارند سلامت خودادراک پایین‌تری را گزارش داده‌اند. نتیجه‌گیری: نتایج به‌دست آمده از این پژوهش بیان‌کننده این است که محدودیت عملکرد‏، محدودیت فعالیت، اختلال روانی، پایگاه اقتصادی اجتماعی‏، تحصیلات و سن پیش‌بینی‌کننده‌های قابل اعتمادی برای سلامت خود ادراک در سالمندان می‌باشند.

Title: بررسی میزان ناتوانی و عوامل مرتبط با آن در سالمندان شهر کاشان در سال 1386

http://salmandj.uswr.ac.ir/article-1-89-fa.html

اهداف: مطالعات از افزایش روند سالمندی و ناتوانی های آن در کشورهای در حال توسعه و روند نزولی ناتوانی‌ها در کشورهای غربی گزارش می‌دهند. با وجود این سلامت و وضعیت ناتوانی سالمندان در کشور ما مورد کم‌توجهی قرار گرفته است. این پژوهش با هدف بررسی میزان ناتوانی و برخی عوامل مرتبط با آن در سالمندان کاشان انجام شد. مواد و روش‌ها: پژوهش به صورت تحلیلی مقطعی بر یک نمونه 350 نفری از سالمندان 65 سال و بالاتر شهر کاشان انجام شد. از پرسش‌نامه سازمان بهداشت جهانی برای بررسی ناتوانی سالمندان (WHO-DAS-II) استفاده شد. این پرسشنامه دارای 48 سوال است که هر فرد می تواند نمره ای بین 0 تا 144 از آن کسب کند. برای بررسی ارتباط ناتوانی با خصوصیات سالمندان از آمار تحلیلی t و تحلیل واریانس استفاده شد. یافته‌ها: %‌61 واحدهای پژوهش را مردان تشکیل دادند. %12 از سالمندان به تنهایی زندگی می‌کردند. یک چهارم سالمندان سیگار یا مواد اعتیادآور مصرف می‌کردند. اکثر آن‌ها بی‌سواد یا دارای سواد ابتدایی بودند. بیش از دو سوم فاقد فعالیت منظم فیزیکی بودند. %20 سالمندان ناتوانی متوسط و %3/4 ناتوانی شدید داشتند. ارتباط معنی‌داری میان شدت ناتوانی با سن، جنس، شیوه زندگی، نیاز به کمک، وضعیت تأهل، محل سکونت، اعتیاد، توانایی کارکردن، فعالیت منظم بدنی، سطح سواد و نیز ابتلا به چند بیماری مشاهده شد. نتیجه‌گیری: سالمندان مطالعه حاضر در مقایسه با سایر کشورها از ناتوانی کمتری برخوردار بودند اما با توجه به روند افزایش جمعیت سالمندان، توجه به سلامت کاهش ناتوانی‌های آن‌ها ضروری است. بیشتربودن شدت ناتوانی در زنان و افراد کم‌سواد ضرورت توجه به این گروه‌های آسیب‌پذیر را نشان می‌دهد.

Title: مقایسه احساس تنهایی و سلامت عمومی در سالمندان ساکن خانواده و سرای سالمندان مناطق شمال شهر تهران

http://salmandj.uswr.ac.ir/article-1-90-fa.html

اهداف: با توجه به رشد سریع جمعیت سالمندان در قرن 21 به ویژه در کشورهای در حال توسعه، توجه به برآورده‌کردن نیازهای جسمانی و روانی سالمندان ضروری است. پژوهش حاضر به منظور مقایسه احساس تنهایی و سلامت عمومی در سالمندان ساکن خانواده و سرای سالمندان مناطق شمال شهر تهران انجام شده است. مواد و روش‌ها: مطالعه از نوع مقطعی-مقایسه‌ای بوده است. نمونه‌های پژوهش 180 نفر (96 سالمند مرد و 84 سالمند زن؛ 103 سالمند ساکن خانه سالمندان و 77 سالمند ساکن در خانواده) با میانگین سنی 73 سال و انحراف استاندارد 8 بود که به شیوه در دسترس انتخاب شدند. پرسش‌نامه‌های سلامت عمومی و احساس تنهایی در مورد آن‌ها به اجرا درآمد و داده‌ها با استفاده از تحلیل واریانس دو عاملی مورد تحلیل قرار گرفت. یافته‌ها: یافته‌های پژوهش نشان داد که بین سالمندان ساکن خانه سالمندان و سالمندان ساکن در خانواده‌ها از لحاظ سلامت عمومی تفاوت معنی‌داری وجود دارد. (F(1/176)=105/5, P=0/0001) به این صورت که سلامت عمومی سالمندان ساکن خانه سالمندان به‌طور معنی‌داری کمتر از سالمندان ساکن خانواده‌ها بود. همچنین یافته‌های پژوهش نشان داد که بین سالمندان ساکن خانه سالمندان و سالمندان ساکن خانواده‌ها از لحاظ احساس تنهایی تفاوت معنی‌داری وجود دارد. (P=0/0001 و 5/105=(1/176)F) به این صورت که نمرات احساس تنهایی سالمندان ساکن خانه سالمندان به طور معنی‌داری بیشتر از سالمندان ساکن خانواده‌ها بود. علاوه بر این یافته‌های تحقیق نشان داد که بین احساس تنهایی مردان و زنان تفاوت معنی‌داری وجود دارد، (F(1/176)=15/84, P=0/0001) بدین صورت که احساس تنهایی مردان به‌طور معنی‌داری بیشتر از زنان بود. نتیجه‌گیری: نتایج پژوهش نشان داد که زندگی با سایر اعضای خانواده بر سلامت جسمانی و روانی سالمندان تأثیر مثبت دارد. بنابراین با توجه به موقعیت روانی‌فرهنگی سالمندان در ایران، بهترین مکان برای برآورده‌شدن نیازهای روانشناختی آنان خانواده است.

Title: تأثیر شش هفته برنامه تمرین عملکردی بر تعادل ایستا و پویای مردان سالمند سالم

http://salmandj.uswr.ac.ir/article-1-91-fa.html

اهداف: تعادل به عنوان شاخصی برای تعیین میزان استقلال سالمندان در انجام فعالیت های روزمره محسوب می شود. هدف اصلی از انجام این پژوهش بررسی تاثیر یک دوره تمرین عملکردی برتعادل ایستا و پویای مردان سالمند سالم بود. مواد و روش‌ها: 30 مرد سالمند (سن: 3±70/83 سال، وزن: 2/44±70/60 کیلوگرم، قد: 2/28±1/78 متر) که از سلامت عمومی برخوردار بودند، به‌طور داوطلبانه در این تحقیق شرکت کردند که به صورت تصادفی به دو گروه تجربی و کنترل تقسیم شدند. از آزمودنی‌ها یک روز قبل از شروع تمرینات توسط تست‌های شارپند رومبرگ (تعادل ایستا با چشمان باز و بسته) و زمان برخاستن و رفتن (تعادل پویا) به عمل آمد. گروه تجربی به انجام یک دوره تمرین عملکردی شش هفته‌ای (سه جلسه در هفته) پرداختند و گروه کنترل فعالیت‌های روزانه خود را انجام داد. پس از اتمام تمرینات از دو گروه پس‌آزمون به عمل آمد. از آمار توصیفی برای محاسبه میانگین و انحراف استاندارد اطلاعات و برای تعیین اختلاف بین پیش آزمون و پس آزمون بین و درون گروهی به ترتیب از t مستقل و وابسته در سطح معنی‌داری (α≤0/05) استفاده شد. یافته‌ها: نتایج نشان داد که بین دو گروه تجربی و کنترل در تست‌های سه‌گانه تعادل ایستا و پویا در پیش‌آزمون اختلاف معنی‌‌داری وجود ندارد اما در پس آزمون، گروه تجربی عملکرد بهتری نسبت به گروه کنترل داشت. نتایج آزمون t همبسته نیز اختلاف معنی‌داری بین پیش و پس‌آزمون گروه تجربی در هر سه تست تعادلی را نشان داد در حالی که بین پیش و پس‌آزمون گروه کنترل تفاوت معنی‌داری مشاهده نشد. نتیجه گیری: نتایج به دست آمده از این تحقیق، موید نقش تمرینات عملکردی در بهبود تعادل ایستا و پویای افراد سالمند بود که به علت عدم بهبود تعادل آزمودنی های گروه کنترل، می‌توان بهبود تعادل گروه تجربی را به اثر این تمرینات نسبت داد، اما با توجه به کوتاهی دوره تمرینی (شش هفته) مطالعات تکمیلی دراز مدت برای بررسی ماندگاری اثرات و پیامدهای بلند مدت این تمرینات ضروری است.

Title: بررسی ارتباط حمایت خانواده و کنترل قندخون در سالمندان مبتلا به دیابت نوع 2

http://salmandj.uswr.ac.ir/article-1-92-fa.html

اهداف: از آنجائی که بخش عمده‌ای از درمان دیابت در منزل انجام می‌شود، خانواده نقش مهمی در مراقبت از این بیماران دارند. هدف از این مطالعه بررسی ارتباط حمایت خانواده و کنترل قندخون در سالمندان مبتلا به دیابت نوع 2 بود. مواد و روش‌ها: این پژوهش از نوع توصیفی- همبستگی بود و تعداد 150 نفر از سالمندان مبتلا به دیابت نوع2 مراجعه کننده به انستیتو غدد درون‌ریز و متابولیسم وابسته به دانشگاه علوم پزشکی ایران جامعه این پژوهش را تشکیل دادند. نمونه‌گیری به صورت غیرتصادفی و در دسترس بود. پرسش‌نامه مورد استفاده شامل سه بخش مشخصات دموگرافیکی نمونه‌ها، تعیین میزان همو‌گلوبین‌گلیکوزیله و تعیین میزان حمایت دریافتی و درک شده بیماران از جانب خانواده با استفاده از ابزار استاندارد "حمایت اجتماعی از بیماران دیابتی-مختص خانواده"بود. داده‌ها با استفاده از آزمون آماری کای‌دو و پیرسون از طریق نرم‌‌ افزار SPSS version-15 تجزیه و تحلیل شد. یافته‌ها: نتایج ارتباط آماری معنی‌داری را بین حمایت خانواده و کنترل قندخون نشان داد (r=-0/56, P<0/0001). همچنین یافته‌ها نشان‌دهنده وجود ارتباط معنی‌دار بین حمایت خانواده و متغیرهای جنس و وضعیت تأهل (P<0/0001) بود. کنترل قند خون نیز با وضعیت تأهل (P=0/02)، وضعیت اقتصادی (P=0/04) و سطح تحصیلات (P=0/05) ارتباط معنی‌داری داشت. نتیجه‌گیری: یافته‌های این پژوهش تاکید مجددی بر نقش حمایت خانواده در بهبود سطح سلامت سالمندان بود. براساس یافته‌های این پژوهش طرح مداخلات پرستاری خانواده محور و مشارکت تمامی اعضاء خانواده در مراقبت از سالمندان مبتلا به دیابت پیشنهاد می‌شود.

Title: تاثیر زانوبند نرم بر تعادل ایستا و پویا در مردان سالمند

http://salmandj.uswr.ac.ir/article-1-93-fa.html

اهداف: حمایت کننده مفصلی موجب فراهم شدن اطلاعات حس عمقی می­‌گردد. این اطلاعات برای حفظ تعادل مورد نیاز می‌باشند. هدف از این مطالعه تعیین اثر استفاده از حمایت‌کننده مفصلی زانو بر تعادل ایستا و پویا در مردان سالمند می­‌باشد. مواد و روش‌ها: در این مطالعه کار آزمایی تصادفی بالینی 22 نفر سالمند در دو گروه مداخله و کنترل شرکت داده شدند. نوع مداخله پوشیدن زانوبند الاستیکی نرم بود. آزمون­‌های مورد استفاده عبارت بودند از: آزمون ایستادن با پای کنار هم، آزمون ایستادن تندوم، آزمون ایستادن روی یک پا که به عنوان آزمون­‌های تعادلی استاتیک در یکی از سه شرایط چشم باز و روی سطح سفت، چشم بسته و روی سطح سفت و چشم بسته روی سطح نرم اجرا شدند. آزمون­‌های تعادلی داینامیک شامل آزمون پنج بار نشستن و برخاستن از روی صندلی، آزمون راه رفتن سرعتی (در دو نوبت)، آزمون دستیابی (با چشم باز و بسته) و آزمون قدم زدن در مربع‌ها بود. به منظور بررسی تفاوت بین کارایی آزمون‌های تعادلی قبل و بعد از مداخله در دو گروه از تحلیل واریانس اندازه‌گیری مکرر استفاده شد. یافته‌ها: نتایج نشان داد که تفاوت بین نتایج آزمون‌های استاتیک و داینامیک در مراحل مختلف در گروه مداخله و کنترل معنی‌دار نیست. در مقایسه بین دو گروه مداخله و کنترل نیز آزمون‌ها تفاوت دو گروه با یکدیگر معنی‌دار نبود. نتیجه‌گیری: حمایت‌کننده مفصلی زانو نمی‌­تواند موجب بهبود کارایی عملکردهای تعادلی ایستا و پویا در مردان سالمند گردد. لذا تجویز آن با اهداف دیگر اختلال در کارایی تعادلی فرد ایجاد نمی­‌نماید.

Title: اپیدمیولو‍‍ژی سرطان‌های دستگاه گوارش در سالمندان استان آذربایجان شرقی در سال 1385

http://salmandj.uswr.ac.ir/article-1-94-fa.html

اهداف: کانسرهای دستگاه گوارش در سالمندان جزء شایعترین کانسرها در سراسر جهان است. با توجه به شیوع بالای کانسرهای دستگاه گوارش در این گروه سنی، در این مطالعه بر آن شدیم تا میزان بروز کانسرهای دستگاه گوارش را در افراد سالمند (65 سال و بالاتر) به دست آورده و برای بررسی تاثیر افزایش سن در بروز این کانسرها، میزان بروز این گروه سنی را با گروه سنی افراد میانسال (45 تا 64 سال) و گروه سنی جوان ( 15 تا 45 سال) مقایسه کنیم. مواد و روش‌ها: در یک مطالعه مقطعی تمامی موارد جدید مبتلا به کانسرهای دستگاه گوارش در استان آذربایجان شرقی- ایران در طول مدت یکسال از تمامی مراکز آموزشی و درمانی، بیمارستان‌ها، مراکز پاتولوژی، مراکز شیمی درمانی، مرکز رادیوتراپی، مراکز آندوسکوپی، بیماران کانسری مصرف‌کننده اپیویید و لیست متوفیان گورستان رسمی تبریز ثبت گردید. یافته‌ها: در طول مدت یکسال از 1533 بیمار مبتلا به کانسر دستگاه گوارش، 824 بیمار (%53/8)در گروه سنی بالای 65 سال، 522 بیمار (%34/1) در گروه سنی میانسال و 125 بیمار (%8/2) در گروه سنی جوان ثبت گردید.%4 از بیماران نیز سن نامشخص داشتند. در گروه سنی سالمندان، %67/2 از بیماران جنس مذکر و %32/8 از جنس مونث بودند. میانگین سنی در زمان تشخیص برای مردان  5/64±73/74 سال و برای زنان 5/88±73/39 سال بود. بر اساس میزان بروز سنی استاندارد شده (ASR)، کانسر معده با بروز 222/30 در میان مردان سالمند و کانسر مری با بروز 83/13 در میان زنان سالمند شایع‌ترین کانسر دستگاه گوارش بود. میزان بروز کانسر معده در مردان سالمند تقریباٌ 3 برابر بیشتر از میزان بروز آن در زنان سالمند (80/81=ASR) به دست آمد. کانسر کولورکتال با میزان بروز 63/02 و 43/51 به ترتیب در مردان و زنان سالمند، در رتبه سوم قرار گرفت. کانسر روده کوچک (بدون درنظر گرفتن کانسر آنال) کمترین ASR را در مردان و زنان سالمند (به ترتیب 3/42 و 3/59) به خود اختصاص می داد. نتیجه‌گیری: در کشور ما، به دلیل ارتقای سطح سلامت و نیز افزایش مراقبت‌های دوران سالمندی نسبت به سابق، میزان امید به زندگی و کیفیت زندگی در سالمندان در حال افزایش است. همچنین با توجه به بروز بالای کانسردر جمعیت سالمند و نیز میزان بالای جمعیت جوان که در چندین سال آینده به سمت جمعیت سالمندی سوق پیدا خواهند کرد، در آینده بسیار نزدیک، کانسر به عنوان یکی از بزرگترین مشکلات بهداشتی و تهدیدکننده سلامت خواهد بود. بنابراین پیشگیری از سرطان با استفاده از روش‌های غربالگری و شناسایی بیماران در مراحل ابتدایی بیماری به خصوص در جمعیت سالمند شایسته توجه فوری و انجام اقدامات ضروری است.

Title: وضعیت فعالیت‌های اجتماعی ونحوه گذران اوقات فراغت در سالمندان ایران و سوئد

http://salmandj.uswr.ac.ir/article-1-95-fa.html

اهداف: در میان عوامل اجتماعی تعیین کننده سلامت، فرهنگ و قومیت در تعریف نیازهای بهداشتی اقشار مختلف جمعیتی نقشی کلیدی دارد. هدف از انجام این پژوهش شناسائی و مقایسه وضعیت فعالیت های اجتماعی و نحوه گذران اوقات فراغت سالمندان ایرانی، سوئدی و ایرانی مهاجر مقیم کشور سوئد بوده است. مواد و روش‌ها: مطالعه حاضر مقطعی واز نوع توصیفی-تحلیلی است. شهر تهران در ایران و شهر استکهلم در سوئد به عنوان محیط پژوهش انتخاب گردید. جمعیت مورد بررسی سالمندان ایرانی مقیم منزل ساکن ایران و سوئد (مهاجرین ایرانی مقیم سوئد) و سالمندان سوئدی مقیم منزل ساکن سوئد بودند که توانایی برقراری ارتباط و پاسخگویی به سؤالات پرسش‌نامه را داشته ومایل به شرکت در مطالعه بودند. 825 سالمند ایرانی به روش نمونه گیری تصادفی خوشه‌ای با رعایت نسبت جنسی از مناطق مختلف شهر تهران، 305 سالمند سوئدی و نیز 101 سالمندایرانی مقیم سوئد از طریق اداره آمار شهر استکهلم وارد مطالعه شدند. ابزار چمع‌آوری داده‌ها، پرسش‌نامه "بررسی نیازها و سلامت اجتماعی" بود که توسط محققان این مطالعه و در هشت محور مختلف ساخته شد. روایی صوری و محتوائی پرسش‌نامه با استفاده از نظرات صاحب‌نظران حوزه سالمندی و گروه هدف و پایائی آن با محاسبه آلفای کرونباخ و روش آزمون-باز آزمون ارزیابی شد. در تحلیل آماری داده‌ها از روش‌های برآورد و رگرسیون لجستیک استفاده شد. یافته‌ها: یافته‌های این مطالعه شامل نتایج حاصل از ارزیابی وضعیت فعالیت‌های اجتماعی و نحوه گذران اوقات فراغت و مقایسه این ابعاد در سالمندان ساکن منزل در دو کشور ایران و سوئد بود. آزمودنی‌ها در هر سه گروه به دامنه سنی 60 تا 77 سال تعلق داشته و عمدتا متأهل بودند. نتایج نشان داد که به رغم اشتراکات بسیار، فراوانی و نوع فعالیت‌های اجتماعی و ایام فراغت در سه گروه فوق به دلیل تفاوت‌های فرهنگی و محیطی مختلف است. عواملی مانند گسترش شهرنشینی، کمبود تسهیلات، عدم آشنایی یا توجه به شیوه زندگی سالم سبب شده است که میزان رضایت شهروندان سالمند ایرانی از روابط اجتماعی و نحوه گذران ایام فراغت از سالمندان سوئدی کمتر باشد. از سوی دیگر، توجه به بعد معنوی سلامت در سالمندان ایرانی قوی‌تر بوده وبا سالمندان سوئدی اختلاف معنی‌دار دارد در حالی که در مورد فعالیت‌های گروهی این رابطه کماکان معنی دار ولی معکوس می‌شود (P=0/000). همچنین، توجه ناکافی به تحرک فیزیکی و ورزش، خصوصا در زنان سالمند ایرانی از نکاتی است که باید مورد عنایت برنامه ریزان و سیاست گذاران عرصه بهداشت کشور قرار گیرد. نتیجه‌گیری: نتایج به دست آمده در این پژوهش بر لزوم انجام نیاز سنجی با رویکرد فرهنگی با استفاده از ابزارهای مناسب معتبر و پایا تاکید دارد. ابزار ساخته شده در روند انجام این مطالعه که اعتبار و پایایی آن به اثبات رسید می‌تواند در بررسی سیستم‌ها و خدمات بهداشتی-مراقبتی درون فرهنگی و میان فرهنگی مورد استفاده قرار گیرد.
